# Exploiting a wheat EST database to assess genetic diversity

**DOI:** 10.1590/S1415-47572010005000094

**Published:** 2010-12-01

**Authors:** Ozge Karakas, Filiz Gurel, Ahu Altinkut Uncuoglu

**Affiliations:** 1 The Scientific and Technological Research Council of Turkey, Marmara Research Center, Genetic Engineering and Biotechnology Institute, Gebze-KocaeliTurkey; 2 Department of Molecular Biology and Genetics, Istanbul University, IstanbulTurkey

**Keywords:** biodiversity, EST, genetic diversity, *Triticum*, yellow rust

## Abstract

Expressed sequence tag (EST) markers have been used to assess variety and genetic diversity in wheat (*Triticum aestivum*). In this study, 1549 ESTs from wheat infested with yellow rust were used to examine the genetic diversity of six susceptible and resistant wheat cultivars. The aim of using these cultivars was to improve the competitiveness of public wheat breeding programs through the intensive use of modern, particularly marker-assisted, selection technologies. The F_2_ individuals derived from cultivar crosses were screened for resistance to yellow rust at the seedling stage in greenhouses and adult stage in the field to identify DNA markers genetically linked to resistance. Five hundred and sixty ESTs were assembled into 136 contigs and 989 singletons. BlastX search results showed that 39 (29%) contigs and 96 (10%) singletons were homologous to wheat genes. The database-matched contigs and singletons were assigned to eight functional groups related to protein synthesis, photosynthesis, metabolism and energy, stress proteins, transporter proteins, protein breakdown and recycling, cell growth and division and reactive oxygen scavengers. PCR analyses with primers based on the contigs and singletons showed that the most polymorphic functional categories were photosynthesis (contigs) and metabolism and energy (singletons). EST analysis revealed considerable genetic variability among the Turkish wheat cultivars resistant and susceptible to yellow rust disease and allowed calculation of the mean genetic distance between cultivars, with the greatest similarity (0.725) being between Harmankaya99 and Sönmez2001, and the lowest (0.622) between Aytin98 and Izgi01.

## Introduction

Wheat (*Triticum aestivum* L.) is one of the most important crops in the world and is grown in all agricultural regions of Turkey. The total area cultivated worldwide and in Turkey is 210 and 9.4 million ha, respectively (Zeybek and Yigit, 2004). The allohexaploid wheat genome (2n = 6x = 42) is one of the largest among crop species, with a haploid size of 16 billion bp (Bennett and Leitch, 1995), and its genetics and genome organization have been extensively studied using molecular markers (Yu *et al.*, 2004; Ercan *et al.*, 2010; Akfirat-Senturk *et al.*, 2010).

PCR-based molecular markers such as simple sequence repeats (SSR) (Plaschke *et al.*, 1995)_,_ restriction fragment length polymorphism (RFLP) (Nagaoka and Ogihara, 1997), amplified fragment length polymorphism (AFLP) (Gülbitti-Onarici *et al.*, 2007), selective amplification of microsatellite polymorphic loci (SAMPL) (Altintas *et al.*, 2008) and random amplified polymorphic DNA (RAPD) (Asif *et al.*, 2005) are easy to use and show a high degree of polymorphism. A number of wheat genetic maps have been constructed using PCR based markers (Li *et al.*, 2007).

In recent year, expressed-sequence tags (ESTs) have become a valuable tool for genomic analyses and are currently the most widely used approach for sequencing plant genomes, both in terms of the number of sequences and total nucleotide counts (Rudd, 2003). EST analysis provides a simple strategy for studying the transcribed regions of genomes, and renders complex, highly redundant genomes such as that of wheat amenable to large-scale analysis. The number of ESTs and cDNA sequences in public databases such as GenBank has increased exponentially in recent few years, and EST-based markers have been used to distinguish varieties and assess genetic diversity in wheat (Kantety *et al.*, 2002; Leigh *et al.*, 2003).

Yellow rust, a destructive disease of wheat triggered by the biotrophic fungus *Puccinia striiformis* f. sp*. tritici* (Chen 2005), is the most frequent and important cereal disease in Turkey, where it causes grain yield losses of 40%-60% and lowers the quality of cereal products (Zeybek and Yigit, 2004). In this study, an EST database for yellow rust-infested wheat was used, in conjunction with a multi-variate statistical package (MVSP v.3.1), to assess the genetic diversity of yellow rust resistant and susceptible wheat genotypes. For this, EST sequences were assembled into longer contiguous sequences (contigs) using Vector NTI 10.0 software. Difficulties related to sequencing errors and the determination of orthology associated with the use of ESTs for systematics can be minimized by using several reads to assemble contigs and EST clusters for each region (Parkinson *et al.*, 2002; Torre *et al.*, 2006). The knowledge gained about the genetic constitution and relationships of genotypes using this approach should prove useful in the optimization of wheat breeding programs.

## Materials and Methods

###  Plant material and evaluations

Six homozygous bread wheat genotypes (three yellow rust-resistant cultivars: PI178383, Izgi01, Sönmez2001, and three yellow rust-susceptible cultivars: Harmankaya99, ES14, Aytin98) were obtained from the Anatolian Agricultural Research Institute, Eskisehir, Turkey. The resistance of the parental cultivars and F_2_ generation was tested in greenhouses by applying uredospores. Two weeks after the inoculation the infection was scored on a scale of 0-9 (McNeal *et al.*, 1971), with scores of 0-6 indicating a low infection and 7-9 indicating a high infection. The disease score for PI178383, Izgi01 and Sönmez2001 was 0 while that of Harmankaya99, ES14 and Aytin98 was 8, this confirming the resistance and susceptibility of the parental genotypes.

###  Analysis of wheat yellow rust ESTs

**Figure 1:**
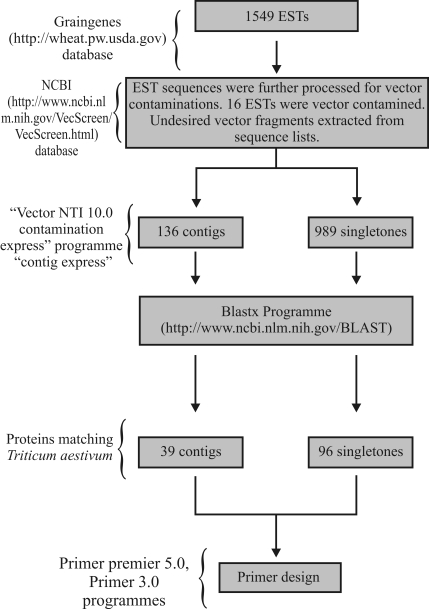
Schematic overview of the strategy for using the EST database and exploiting contigs and singletons.

ESTs from a yellow rust-infected wheat cDNA library (TA117G1X) were selected from the GrainGenes website and processed by means of VecScreen database searches to remove undesired vector fragments from the sequences. The Vector NTI 10.0 contig express program (InforMax, Bethesda, MD, USA) was used to construct contig tags from the EST sequences and the Contig Express module was used to assemble small fragments in text or chromatogram formats into contigs (Lu and Moriyama, 2004). Singletons were constructed from unassembled ESTs. The EST sequences were aligned and analyzed with ClustalW v.1.82 to identify conserved domains. Functional annotation was done using the BlastX algorithm of the Basic Alignment Search Tool (Altschul *et al.*, 1990). PCR primers for the contigs and singletons selected for further characterization were designed with Primer Premier 5.0 and Primer 3.0 software (Figure 1). EST-derived contig and singleton primers were used to assess the genetic diversity of the six wheat genotypes.

###  PCR analyses of contigs and singletons

Total genomic DNA was extracted from the leaves of resistant and susceptible plants using the method of Weining and Langridge (1991) as modified by Song and Henry (1995). Genomic DNA amplifications with sense and antisense primers were done using a PTC-100 MJ thermocycler (MJ Research, Watertown, MA) in a 25 μL reaction volume. Each reaction contained 1X *Taq* buffer (MBI Fermentas, Germany), 2.5 mM MgCl_2_ (MBI Fermentas), 0.2 mM dNTP (MBI Fermentas), 400 nM of forward primer, 400 nM of reverse primer, 0.625 U of *Taq* polymerase/μL (MBI Fermentas) and 100 ng of genomic DNA. The thermal cycling parameters were: 3 min at 94 °C (initial denaturation), 37 cycles of 1 min at 94 °C, 1 min at 40-58 °C (depending on the annealing temperature) and 1 min at 72 °C, followed by a final extension at 72 °C for 10 min. PCR products were separated in 2% agarose gels, stained with ethidium bromide and examined under UV light.

###  Genetic similarity estimation and cluster analyses

Each contig and singleton band was scored as absent (0) or present (1) for the different cultivars and the data were entered into a binary matrix as discrete variables (‘1' for presence and ‘0' for absence of a homologous fragment). Only distinct, reproducible, well-resolved fragments were scored and the data were analyzed using MVSP 3.1 software (Kovach, 1999). This software package was also used to calculate Jaccard (1908) similarity coefficients to construct a dendrogram by a neighbour-joining algorithm.

## Results

###  Assembly of contigs and blast analysis

**Figure 2:**
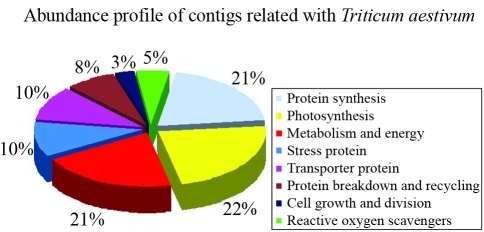
Classification of contigs homologous to proteins of known function.

**Figure 3:**
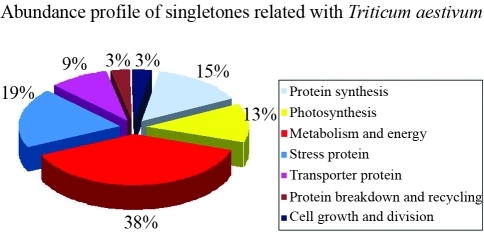
Classification of singletons homologous to proteins of known function.

Table 1 summarizes the characteristics of the database used in this analysis. 1549 ESTs were selected from a yellow rust-infested wheat cDNA library (TA117G1X) and used to assemble 136 contigs. The number of individual ESTs belonging to each contig ranged from 2 to 57. Singletons were derived from unassembled ESTs and accounted for 72.63% of ESTs. Tables 2  and 3 show the results of the NCBI database searches done using the contig and singleton sequences. The BlastX searches revealed that 39 contigs (29%) were homologous to wheat genes (Figure 2). Contigs 3, 4, 11, 13, 16 and 112 did not match any organism. Contig 77 matched a sequence of unknown function (data not shown) while other contigs (71%) showed homology to genes of known function. The BlastX search also showed that 96 singletons (10%) were homologous to wheat genes (Figure 3), whereas 147 singletons (14%) did not match any organism and had no functional annotation (data not shown). The 39 contigs and 96 singletons that matched wheat proteins were assigned to eight functional groups that included protein synthesis, photosynthesis, metabolism and energy, stress proteins, transporter proteins, protein breakdown and recycling, cell growth and division and reactive oxygen scavengers. Photosynthesis was the major functional category of contigs, with nine proteins (22%), whereas cell growth and division was the smallest, with one protein (3%) (Figure 2). Metabolism was the major functional category of singletons, with 37 proteins (38%), whereas protein breakdown and recycling and cell growth and divison were the smallest functional categories, with three proteins (3%) (Figure 3). Tables 4  and 5 show the sense and antisense primers used to assess the genetic diversity of wheat cultivars; these primers were designed based on the contig and singleton sequences that were homologous to wheat genes.

###  EST-derived contig and singleton polymorphisms

**Figure 4:**
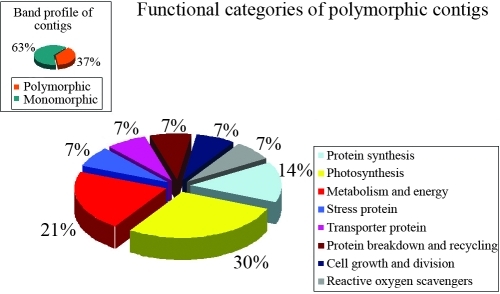
Functional categories of polymorphic contigs.

**Figure 5:**
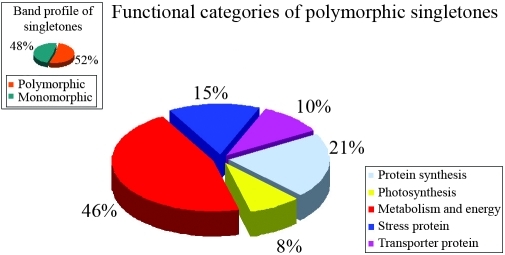
Functional categories of polymorphic singletons.

PCR analyses with the contig and singleton primers showed that the most polymorphic functional categories were photosynthesis (30%) and metabolism and energy (46%) for contigs and singletons, respectively (Figures 4  and 5). Of the 39 contig and 92 singleton primers used to characterize the genetic diversity of the six wheat genotypes, 14 contig and 48 singleton primers were polymorphic in susceptible and resistant wheat cultivars. Table 6 summarizes the mean genetic distance and genetic identity between the cultivars as determined by MVSP 3.1. Pairwise within-group distances ranged from 0 to 0.725, with the highest similarity (0.725) occurring between Harmankaya99 and Sönmez2001 and the lowest (0.622) between Aytin98 and Izgi01.

**Figure 6:**
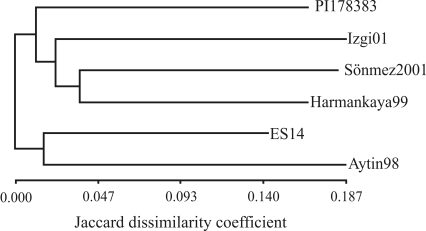
Dendrogram based on the genetic similarity of six Turkish bread wheat (*Triticum aestivum* L.) genotypes.

Figure 6 shows the dendrogram based on the similarity index (Jaccard's coefficient) of the six cultivars. Two main clusters were observed, the first of which included cultivars Aytin98 and ES14 while the second was divided into two subclusters, the first of which comprised PI178383 while the second contained Izgi01, Sönmez2001 and Harmankaya99. The latter subcluster consisted a group containing Izgi01 and another containing Sönmez2001 and Harmankaya99. The construction of this dendrogram demonstrates the ability of EST-derived contigs and singletons in detecting extensive genetic diversity in genotypes with an expected narrow genetic pool.

## Discussion

Genome-marker technologies are particularly valuable for analyzing crops, such as wheat, that have relatively low levels of genetic diversity (Plaschke *et al.*, 1995). DNA markers such as AFLP (Gülbitti-Onarici *et al.*, 2007), RAPD (Asif *et al.*, 2005), EST-SSR (Leigh *et al.*, 2003), SSRs (Chen*,* 2005) and internal transcribed spacer (ITS) (Zhang *et al.*, 2002) are the most convenient data sources. EST databases represent a potentially valuable resource for developing molecular markers for evolutionary studies. Since EST-derived markers come from transcribed regions of the genome they are likely to be conserved across a broader taxonomic range than other types of markers (Pashley *et al.*, 2006).

The low level of genetic diversity expected between self-pollinating plants means that EST databases can be useful tools for genetic studies in wheat and related species. Our results indicate that EST-derived primers were good tools for assessing the genetic diversity in wheat cultivars. A relatively high level of polymorphism (58.61% of loci were polymorphic) was observed with 39 contig and 92 singleton primers across the six wheat genotypes, despite the fact that all of them were local cultivars from geographically close locations. Several other studies have reported polymorphism in self-pollinating plants, including tef (4%) (Bai *et al.*, 1999), azuki (18%) (Yee *et al.*, 1999), rice (22%) (Maheswaran *et al.*, 1997), sugar beet (50%) (Schondelmaier *et al.*, 1996) and wild barley (76%) (Pakniyat *et al.*, 1997). In a work similar to that reported here, Wei *et al.* (2005) used microsatellite markers to assess the polymorphic divergence in wheat landraces highly resistant to *Fusarium* head blight (FHB). The level of polymorphism observed among 20 wheat landraces resistant to FHB and four wheat landraces susceptible to FHB was 97.5% with a mean genetic similarity index among the 24 genotypes of 0.419 (range: 0.103 to 0.673).

In conclusion, we have used an EST database to examine the genetic diversity among Turkish wheat cultivars resistant and susceptible to yellow rust disease. Our results indicate that EST databases can be used to assess genetic diversity and identify suitable parents in populational studies designed to detect genes related to disease resistance.

## Figures and Tables

**Table 1:** General characteristics of ESTs from yellow rust-infested wheat (*Triticum aestivum*).

Database characteristics	
Library name	TA117G1X
Stage	-
Total number of ESTs	1,549
Contigs	136
Total contig size (bp)	80,241
Unigenes	1,125 (72.6%)
EST contigs	560
Singletons	989 (63.8%)
Contaminated ESTs	16

**Table 2:** Contigs that showed homology to genes with proteins matching *Triticum aestivum* identified in a BlastX search of the NCBI database.

Contig name	Blast hit number	Annotation	Accession number
Contig 1	100	ribosomal protein L16	NP_114295
Contig 8	44	ribosomal protein S7	AAW50993
Contig 9	101	lipid transfer protein	ABB90546
Contig 12	101	chlorophyll a/b binding protein, chloroplast precursor (LHCII type I CAB) (LHCP)	P04784
Contig 17	100	ferredoxin, chloroplast precursor	P00228
Contig 19	100	triosephosphate-isomerase	CAC14917
Contig 21	196	putative glycine decarboxylase subunit	AAM92707
Contig 22	281	eukaryotic translation initiation factor 5A1	AAZ95171
Contig 24	100	single-stranded nucleic acid binding protein	AAA75104
Contig 30	100	cytosolic glyceraldehyde-3-phosphate dehydrogenase	AAP83583
Contig 33	294	chlorophyll a/b-binding protein WCAB precursor [*Triticum aestivum*]	AAB18209
Contig 34	65	jasmonate-induced protein	AAR20919
Contig 35	44	oxygen-evolving enhancer protein 2, chloroplast precursor (OEE2)	Q00434
Contig 39	100	geranylgeranyl hydrogenase	AAZ67145
Contig 40	100	chlorophyll a/b-binding protein WCAB precursor	AAB18209
Contig 46	102	chlorophyll a/b-binding protein WCAB precursor	AAB18209
Contig 49	31	oxygen-evolving complex precursor	AAP80632
Contig 52	9	metallothionein-like protein 1 (MT-1)	P43400
Contig 55	198	glycine-rich RNA-binding protein	BAF30986
Contig 57	100	type 1 non-specific lipid transfer protein precursor	CAH04983
Contig 58	33	RUB1-conjugating enzyme	AAP80608
Contig 63	103	oxygen-evolving enhancer protein 1, chloroplast precursor (OEE1) (33 kDa subunit of oxygen evolving system of photosystem II) (OEC 33 kDa subunit) (33 kDa thylakoid membrane protein)	P27665
Contig 65	101	acidic ribosomal protein P2	AAP80619
Contig 66	199	cyclophilin A-1	AAK49426
Contig 73	190	dehydroascorbate reductase	AAL71854
Contig 75	63	metallothionein	AAP80616
Contig 80	33	wali7	AAC37416
Contig 90	52	putative membrane protein	ABB90549
Contig 91	100	cold shock protein-1	BAB78536
Contig 93	155	Ps16 protein	BAA22411
Contig 96	109	elongation factor 1-alpha (EF-1-alpha)	Q03033
Contig 99	72	histone H1 WH1A.2	AAD41006
Contig 105	131	ribulose-bisphosphate carboxylase (EC 4.1.1.39) small chain precursor (clone pWS4.3) - wheat	RKWTS
Contig 110	82	cytochrome b6-f complex iron-sulfur subunit, chloroplast precursor (Rieske iron-sulfur protein) (plastohydroquinone:plastocyanin oxidoreductase iron-sulfur protein) (ISP) (RISP)	Q7X9A6
Contig 113	103	lipid transfer protein 3	AAP23941
Contig 122	163	ribulose-1,5-bisphosphate carboxylase/oxygenase small subunit	BAB19814
Contig 133	100	ribosomal protein L36	AAW50980
Contig 135	100	60s ribosomal protein L21	AAP80636
Contig 136	100	histone H2A.2.1	P02276

**Table 3:** Singletons showing homology to genes with proteins matching *Triticum aestivum* identified in a BlastX search of the NCBI database.

Singleton name	Blast hit number	Annotation	Accession number
CA599282	199	ATP synthase CF1 alpha subunit	NP_114256
CA599218	88	ribulose-1,5-bisphosphate carboxylase/oxygenase small subunit	BAB19811
CA598725	191	ribosomal protein L14	NP_114294
CA597765	119	RuBisCO large subunit-binding protein subunit alpha, chloroplast precursor (60 kDa chaperonin subunit alpha) (CPN-60 alpha)	P08823
CA597760	100	type 1 non-specific lipid transfer protein precursor	CAH69210
CA597766	3	aintegumenta-like protein	ABB90555
CA597808	116	geranylgeranyl hydrogenase	AAZ67145
CA597830	100	14-3-3 protein	AAR89812
CA597851	49	plastid glutamine synthetase isoform GS2c	AAZ30062
CA597983	100	GRAB2 protein	CAA09372
CA598020	103	protein H2A.5 (wcH2A-2)	Q43213
CA598034	100	histone deacetylase	AAU82113
CA598102	22	WIR1A protein	Q01482
CA598128	100	probable light-induced protein	AAP80856
CA598130	100	tubulin beta-2 chain (beta-2 tubulin)	Q9ZRB1
CA598143	172	thioredoxin M-type, chloroplast precursor (TRX-M)	Q9ZP21
CA598151	100	lipid transfer protein precursor	AAG27707
CA598174	200	S28 ribosomal protein	AAP80664
CA598181	110	pathogenisis-related protein 1.2	CAA07474
CA598182	2	pathogenisis-related protein 1.2	CAA07474
CA598187	98	VER2	BAA32786
CA598196	1	putative cytochrome c oxidase subunit	AAM92706
CA598235	100	plasma membrane intrinsic protein 1	AAF61463
CA598239	151	triosephosphate translocator	AAK01174
CA598244	14	glycosyltransferase	CAI30070
CA598256	100	heat shock protein 80	AAD11549
CA598258	22	fasciclin-like protein FLA26	ABI95416
CA598286	80	elongation factor 1-beta (EF-1-beta)	P29546
CA598296	106	beta-1,3-glucanase precursor	AAD28734
CA598314	11	oxygen-evolving enhancer protein 2, chloroplast precursor (OEE2)	Q00434
CA598347	114	putative ribosomal protein S18	AAM92708
CA598359	198	sucrose synthase type I	CAA04543
CA598366	105	receptor-like kinase protein	AAS93629
CA598421	121	ribulose-bisphosphate carboxylase (EC 4.1.1.39) small chain precursor (clone pWS4.3)	RKWTS
CA598422	75	wali5	AAA50850
CA598432	99	ribosomal protein P1	AAW50990
CA598476	100	LRR19	AAK20736
CA598485	100	ribulose-bisphosphate carboxylase	CAA25058
CA598489	64	histone H2A	AAB00193
CA598518	157	phosphoribulokinase; ribulose-5-phosphate kinase	CAA41020
CA598523	100	ribosomal protein L19	AAP80858
CA598557	79	type 2 non-specific lipid transfer protein precursor	CAH69201
CA598577	252	ferredoxin, chloroplast precursor	P00228
CA598584	258	putative fructose 1-,6-biphosphate aldolase	CAD12665
CA598630	101	translationally-controlled tumor protein homolog (TCTP)	Q8LRM8
CA598637	100	histone H2A	AAB00193
CA598672	100	lipid transfer protein	ABB90546
CA598674	100	glutathione transferase F6	CAD29479
CA598677	100	ribulose-1,5-bisphosphate carboxylase/oxygenase small subunit	BAB19812
CA598687	55	wali6	AAC37417
CA598691	100	type 1 non-specific lipid transfer protein precursor	CAH04983
CA598694	45	cold-responsive LEA/RAB-related COR protein	AF255053
CA598700	195	fructan 1-exohydrolase	CAD48199
CA598719	24	50S ribosomal protein L9, chloroplast precursor (CL9)	Q8L803
CA598755	100	type 1 non-specific lipid transfer protein precursor	CAH69190
CA598762	95	cysteine synthase (O-acetylserine sulfhydrylase) (O-acetylserine (thiol)-lyase) (CSase A) (OAS-TL A)	P38076
CA598818	100	putative fructose 1,6-biphosphate aldolase	CAD12665
CA598837	126	glutathione S-transferase	AAD56395
CA598848	167	glyceraldehyde-3-phosphate dehydrogenase	AAW68026
CA598850	42	putative proteinase inhibitor-related protein	AAS49905
CA598919	43	ferredoxin-NADP(H) oxidoreductase	CAD30024
CA599166	137	cold acclimation induced protein 2-1	AAY16797
CA599172	135	stress responsive protein	AAY44603
CA599235	100	beta-expansin TaEXPB3	AAT99294
CA599238	77	oxygen-evolving enhancer protein 2, chloroplast precursor (OEE2) (23 kDa subunit of oxygen evolving system of photosystem II) (OEC 23 kDa subunit) (23 kDa thylakoid membrane protein)	Q00434
CA599257	101	glyceraldehyde-3-phosphate dehydrogenase	AAW68026
CA599262	196	histone H2A.2.1	P02276
CA599265	2	phosphoglycerate kinase, chloroplast precursor	P12782
CA599271	100	ribosomal protein L18	AAW50985
CA599273	68	outer mitochondrial membrane protein porin (voltage-dependent anion-selective channel protein) (VDAC)	P46274
CA599277	103	putative SKP1 protein	CAE53885
CA599285	154	putative lipid transfer protein	ABB90547
CA598802	100	ribosomal protein L11	AAW50983
CA598930	100	thioredoxin h	CAB96931
CA598940	199	cyc07	AAP80855
CA598941	298	calcium-dependent protein kinase	ABY59005
CA598949	100	putative 40S ribosomal protein S3	AAM92710
CA598961	100	ribosomal protein L13a	AAW50984
CA598962	57	reversibly glycosylated polypeptide	CAA77237
CA598966	282	MAP kinase	ABS11090
CA598975	105	(1,3;1,4) beta glucanase	CAA80493
CA598980	31	minichromosomal maintenance factor	AAS68103
CA599013	100	D1 protease-like protein precursor	AAL99044
CA599015	17	putative beta-expansin	BAD06319
CA599032	114	tonoplast intrinsic protein	ABI96817
CA599049	41	porphobilinogen deaminase	AAL12221
CA599099	100	gamma-type tonoplast intrinsic protein	AAD10494
CA599101	100	small GTP-binding protein	AAD28731
CA599103	19	pre-mRNA processing factor	AAY84871
CA599107	82	sedoheptulose-1,7-bisphosphatase, chloroplast precursor (sedoheptulose bisphosphatase) (SBPase) (SED(1,7)P2ase)	P46285
CA599110	199	ribulose bisphosphate carboxylase small chain PWS4.3, chloroplast precursor (RuBisCO small subunit PWS4.3)	P00871
CA599114	3	metallothionein-like protein 1 (MT-1)	P43400
CA599115	176	type 1 non-specific lipid transfer protein precursor	CAH69199
CA599119	5	putative high mobility group protein	CAI64395
CA599121	51	putative proteinase inhibitor-related protein	AAS49905
CA599135	257	putative cellulose synthase	BAD06322

**Table 4:** Contig primers used for genomic amplifications.

Primer	Sequence (5'-3')	T_a_^o^C	Product size (bp)	Primer	Sequence (5'-3')	T_a_^o^C	Product size (bp)
Contig 1F Contig 1R	ACA gAT AgA AgC Agg ACg AA AAg ggT TgA Agg AAT TAT TgT C	50	370	Contig 58F Contig 58R	ggg CAA gAA gAA ggA AgA gg TgA ggg TTA ggg AAg ggA gA	50	267
Contig 8F Contig 8R	CCT CCA CTT CgC TgC TCC CT gCT CCT ggT TgC CgT TCT CC	53	168	Contig 63F Contig 63R	CAg ggA ggT CgC AAg CAA TCA ACC CAA CgT ACg CAT	48	898
Contig 9F Contig 9R	CAA ACT CgA TAg ggA Tgg C gCT TgA TTT gCA TAT Tgg gAC	50	340	Contig 65F Contig 65R	gCT gCC TAT CTg CTT gCT T CCT TTC TCC Agg gAC CTT T	48	295
Contig 12F Contig 12R	ACg CAC ATC ggA CAC gC CAg CTC CCg gTT CTT gg	53	336	Contig 66F Contig 66R	gCg CAT CgT gAT ggA gCT Tgg gAg CCT TTg TTg TTg g	53	302
Contig 17F Contig 17R	gCC ACC TTC TCA gCC ACA TTC gCC ggA ACA CCA AAC	49	366	Contig 73F Contig 73R	CTg gTT TgC TAC TCC Tgg T Tgg CAT CCT TTg TTC TTT C	46	417
Contig 19F Contig 19R	gCg gCA ACT ggA AAT g AgC CCT TgA gCg gAg T	50	350	Contig 75F Contig 75R	gAg ATg gAC gAg ggA gTg AA ATg ggg TCT CCC TTg TTC TT	50	499
Contig 21F Contig 21R	gCC CTC AAg ATT TCA AgC Ag ggg TTT TCg gAC AgT TTT gA	50	516	Contig 80F Contig 80R	gCC AAg gAg TgA ggA Agg TCg ATT CAC ggA ggA gCA	50	412
Contig 22F Contig 22R	ggA CAC CgA TgA gCA CCA AAg TTg ggA ggT TTC Agg	48	363	Contig 90F Contig 90R	gAT TCg CAT CgC AgC ACA gCg gTT AAA CAg ACC CAg T	50	409
Contig 24F Contig 24R	ggT Tgg CTT CTC CTC CCC T CgA gCT TCC TTg CCg TTC A	51	331	Contig 91F Contig 91R	TTT Tgg TCC TTC ggT TTC g TCC TCC Tgg TgC ggT gA	55	248
Contig 30F Contig 30R	gTT gAT gAg gAC CTT gTT TC TTg TTC ggg ggT TTT ATT TT	44	450	Contig 93F Contig 93R	TTC AgC gAg CAC ggC AAA g gAC ACA Agg ATg gAT ggg A	49	307
Contig 33F Contig 33R	ATg TCC CTC TCC TCg ACC TT AgT ggA TCA CCT CgA gCT TC	51	291	Contig 96F Contig 96R	CTg CTg CTg CAA CAA gAT g gTT CCA ATg CCA CCA ATC T	48	302
Contig 34F Contig 34R	ACT TCC gCA gCC TgT ACC TT CCA ACA ATT AgC CCA CTC AC	53	302	Contig 99F Contig 99R	gCA TCT CCC CTC gAT TCC TA CgA CCC CgC TCT TCT CCT TC	51	250
Contig 35F Contig 35R	CAA Tgg CgT CCA CCT CCT gC AgT CCg gTg ATg gTC TTC TTg g	53	444	Contig 105F Contig 105R	CCg ATA ATA CAA TAC CAT TAC TCC TTT TTT gAC CTC	40	434
Contig 39F Contig 39R	ggT gTT CTA CCg CTC CAA gAC gCC CAT TAC CCT TTT	48	354	Contig 110F Contig 110R	CAT CTC gCT CCC CAC CTT TTT gCC CTT TgT TTg TTT	40	356
Contig 40F Contig 40R	ACC CAC TAT ACC CAg gAg gC TCA gAA Cgg gAA gAA gCA gA	51	338	Contig 113F Contig 113R	CAA AAA TAg CgT gCA Agg Tg TTg TTT CCA gTT Tgg TTg gA	50	304
Contig 46F Contig 46R	gCA Agg Cgg TgA AgA ACg CCC TTT ggA CAg gAA CCC	49	506	Contig 122F Contig 122R	AgC AAg gTT ggC TTC gTC CCg AgA ATT AAC AgC Agg AC	50	474
Contig 49F Contig 49R	CTC gTg CCg AAg ACA gAA A CCC TCC CTT Tgg TTg gTT	48	578	Contig 133F Contig 133R	CgT TAg CAg gAg CgA gTg gAg CAA ATC CAg CgA CCT	49	196
Contig 52F Contig 52R	TTg ggT TCA CAg ATT Tgg Agg gAA gCA ATT AAC Agg gAC ACg	50	432	Contig 135F Contig 135R	gCC gCA CAA gTT CTA CCA Cg ggA TTg ggA gTg ACg gTT CT	51	311
Contig 55F Contig 55R	gAg TAC CgC TgC TTC gTC CCA CCT CCg CCA CTg AA	53	286	Contig 136F Contig 136R	CAC CCA CTC CCA AAC CCT C gAT TTC AAg CAA gAA CCA A	44	337
Contig 57F Contig 57R	CAC ggT TTC CAg CAA gCA TTg gCg TTC Agg gTC CTC	50	227				

**Table 5:** Singleton primers used for genomic amplifications.

Primer	Sequence (5'-3')	T_a_^o^C	Product size (bp)	Primer	Sequence (5'-3')	T_a_^o^C	Product size (bp)
CA598034F CA598034R	AgC CTA AAA AAA AgC ATA Agg AgT CCC gTC AAA AAA	38	418	CA598930F CA598930R	gTg gAC CAT gCA gAT CgA gg ggg ggC AAT TTT TAT TTT Ag	46	366
CA598174F CA598174R	gAC CAA gAA CCg TCT CAT C TCA AgT CTC ACA ACA TCA A	43	263	CA598143F CA598143R	gAT CAA gTg CTg CAA ggT gA TTg TTA TAA Cgg CgC ATC AA	50	279
CA598286F CA598286R	gTT CTC CgA CCT CCA CAC gTC ATC ATC TTC ATC CTT	42	278	CA599110F CA599110R	TCg gCT ACC ACC gTC gCA CC ACC CTC AAT Cgg CCA CAC CT	58	138
CA598347F CA598347R	ggA ACg CAC CTC CTC CCC TC CCA gTC CCg gCA CCT TTg AA	54	326	CA599107F CA599107R	Agg ACA CCA CgA gCA TC CCC CTT ggg AAC AgC Ag	51	241
CA598432F CA598432R	CgC TgA AgA gAA gAA ggA CgC ATA ggA ggA ACC CAC	47	122	CA599218F CA599218R	CCT CCT CTC CTC CgA TAA TA ACA TAg gCA gCT TTC CCA CA	49	420
CA598523F CA598523R	ggA ggA ggA gAg Cgg Cgg C ATA TCC CAg gAg TgA ACg g	50	262	CA598196F CA598196R	ggT CgT TTC gCT CTC CCC ATT TCT CCT CAg CTg gTT	44	158
CA598719F CA598719R	gCC TCA TCC CCC TCC TCC Gc CgA TTC gCT CTT gCT TCC AC	52	266	CA599282F CA599282R	gCg TAg TTC AAg Tgg ggg AAA AAT CAT TTA ggg ggg	42	490
CA598762F CA598762R	ACg gCg ggA Tgg ggg Agg TTT gCT Tgg gAC gAT gAA	44	224	CA598296F CA598296R	gCA CTg CTg gTg gAg ATg gTT Cgg ACg gAT TgA ggC	50	278
CA599271F CA599271R	TCg gCA CgA ggg TAA gAA g AgT TTg gAg CAA Cgg gAg T	49	483	CA598421F CA598421R	CTC CTC TCC TCC gAT AAT A TTg ACC TTC CCT CCC ACC T	47	461
CA598802F CA598802R	gCT CgT CCT CAA CAT CTC TTT CAC CTT CAg gCC ACT	50	214	CA598485F CA598485R	ACC gTT gCT gAC gCT gCC CCC CCA TTg TTC CCC ATT	49	324
CA598725F CA598725R	CAg CgA TAT gCT CgT ATT gg CTC TCA ATT CCT Cgg CAA TC	50	345	CA598584F CA598584R	CCC CTg Agg TgA TTg CTg TCg CCC TTg TAg gTg CCA	50	306
CA599103F CA599103F	TgT CgT CTg CgT ATT ggT g Cgg ACT Tgg TgA CTT gCT A	51	201	CA598818F CA598818R	TCC TTg CTg CCT gCT ACA TCC TCC ATT CTC Cgg TTC	49	362
CA598961F CA598961R	ggA ggA AAA gAg gAA ggA TCA AAT gAg TgT CgC AgA	48	272	CA598677F CA598677R	CgA CTA CCT TAT CCg CTC C ggg TTA CTC CCT TTT TTg A	45	209
CA598949F CA598949R	gTT TgT gAg CgA Tgg CgT TT ATT gAC TTC AgC CTT Tgg gg	51	324	CA598518F CA598518R	TCg gCA CgA ggg AgA AgC ATC ggA Agg Agg TAA AAC	44	444
CA597765F CA597765R	TgA TTT CCT TTA TgC TTg Tg gCT TgT TgC TTg gTg ggg Tg	44	234	CA598700F CA598700R	gAC TCC ATA CAA TCC CCA gCA CCC gTT TTT CCA CAT	47	272
CA598239F CA598239R	ATT CAA CAT CCT CAA CAA gAA ACC CCC AAg gCA CCA	40	372	CA598975F CA598975R	CgC AgT TAg CCA gAg AgA ggA gTT Tgg AgA gCA CgT	51	298
CA598314F CA598314R	ATg gCg TCC ACC TCC TgC TT ggT Tgg TCg ggg TTT gAT TA	50	466	CA598244F CA598244R	ggA gAT ggT Tgg TTg TgT T CCA ggg gTT gTT ggT AAA T	50	378
CA598577F CA598577R	CgA CCT gCC CTA CTC TTg C AAC CCA CCT TgC CTC CAT T	50	125	CA599101F CA599101R	CgT CgT CgC CAC AAg AgT T CgC CCg TgT TCC CCA gAT T	55	363
CA599238F CA599238R	ggC gTC CAC CTC CTg CTT CC TTg TTg TTg ggg TTT gAT TA	44	426	CA597808F CA597808R	CAC CTT CCT CCC TTC CTC CT CAT CTT TgT TgA CCC TCC TT	48	308
CA598919F CA598919R	TAC TgA TTC TTg TgT CTT A CAC CCT TTA TCT ACT TTT A	41	107	CA598837F CA598837R	gAg AgT gAg gAg TgA gAA gA AAA gCA TTA ggg ATT ggA TA	44	436
CA598848F CA598848R	CCA gAT TTC CTT CCC CAT CAg CAC CAg CAg CAg CCC	47	300	CA598850F CA598850R	ACg CCC AgC CCT CAC AAg A ACg gAC CCA CAC ACA AgC A	51	189
CA599257F CA599257R	TgT TCT CAA CCT CCC CTC C CAA CgT ACT CAg CAC CCA g	50	343	CA599262F CA599262R	CCC ACC CAC TCC CAA ACC CT CCg gCC AgC TCC AgC ACC TC	56	266
CA597851F CA597851R	TTT ggA ggC ggC AgA gTA gTC ggT gAA ggg CgT ggT	49	258	CA598020F CA598020R	gTC ACA TCA TCT TCT CCC T TCC CCA ACA TCA ACT CCg T	47	185
CA598130F CA598130R	CTg ggA ggT ggT gTg TgA Tg ACT TTT TTg gTT gAg ggg AA	46	482	CA598235F CA598235R	gCg AgA Agg AAC AgC AAg TTA gAC ggA CCA CgA Agg	49	618
CA598258F CA598258R	CTC TCC CCC CCT CCC CAg gAg TTC ACC CCC gCC CCg	57	338	CA598359F CA598359R	CCC TgC TgA AAT CAT TgT TAg TTg TCg gAg CTC TTg	44	350
CA598637F CA598637R	CAC CTC gTg AgT CCT CgT Cg TgC ggg TCT TCT TgT TgT CC	52	266	CA598674F CA598674R	AAg gTg CTg gAg gTC TAC AAT CAC ggC TTC TTg ggA	47	230
CA599135F CA599135R	AAg gCg AAg AAg CCA ggT TT Tgg ATT ggA ggA TTg ggg AA	53	292	CA599114F CA599114R	CCg Tgg TCg TCC TCg gCg Tg ggC AAT TAC Cgg ggg AAA CT	55	334
CA599099F CA599099R	CTC ggA ggT gAg CgA AAA T gAC CCC CCC gTT gAg AAg C	52	397	CA599049F CA599049R	ATT CTg CTC TgC TCC TCC CAg TTC gTC ACg ggT TTg	51	278
CA599032F CA599032R	gCC gAT CCA TTC ATC CCg A AgC AgT TgC CCC ACC CAg T	56	375	CA599013F CA599013R	TgA ACA AAg gAg ACA Cgg T TAT TgA TTg gAT TAA ggC C	45	235
CA598962F CA598962R	CAg ggA Cgg TgA CTg TgC C AAT gTC gTT TgC ggT TgT A	51	225	CA598940F CA598940R	gAC gCT CAA gCC CCC Ag Agg TTT gTT TgC CCA TA	47	601
CA599166F CA599166R	Agg gCT CCT ATg CTT CgC gTT gTA CgC CgC TTg gTC	54	211	CA599172F CA599172R	gCA gCC gAC ggT gAA gAt gAg ggC gTT gAA gTT TgA gTA g	53	359
CA597830F CA597830R	CgT gAg AAC AgC gAA gCg gAT TgA TgC gAA CAT Agg C	54	331	CA597983F CA597983R	TCA CgC ACT ACC TCA CCC CCC TTC CAg TAC CCT TTC T	52	208
CA598102F CA598102R	ggC ACA gAC CCT AAC CAC gAg TAC ATT CAC ggA gAC g	54	262	CA598181F CA598181R	CAC CCC gCA ggA CTT CgT TTT ATT TCC AgT TgA TTA	36	382
CA598187F CA598187R	TAg TAT TCT CCC CgC CAC CAT CCT TTA ATT TTT TCA	36	450	CA598128F CA598128R	gCC TTC TTg AAC CAT CCT g gCT TTg AAA TTT ggC gCC C	49	451
CA598256F CA598256R	ggg CAT TgT TgA CTC TgA TTg TTC TCg gCA ATC TCA	52	135	CA598366F CA598366R	CCC gTg gCA gTC AAg ATg TTg AAg CCC AAC Agg ATg	54	347
CA598422F CA598422R	CAC gAg TgA AgT gAg AgC TAT TTT ATT TTA ggC ggA	38	356	CA598476F CA598476R	ATT TCC CgA AgT TAg gCg CTC AAg ggC TgT AAg gTg	52	160
CA598630F CA598630R	CAA AgC AAA TCC CAC AAT TgA ggC gTA ACA TCC AAg	52	383	CA598687F CA598687R	gAg CAA gTT TAg gAg CgA CCA A ATg TAC ggg AAg gCg gAg C	53	285
CA598694F CA598694R	AAT gTC Tgg CTg ggT TCA TCA gTC TTT CTT Tgg Tgg C	52	352	CA599121F CA599121R	AAA CAA CCA TgA AgA ACA CC CAC ATC TAC gCA CAA AAA Cg	48	370
CA598966F CA598966R	ggC TgT TTg AgA ATg gAC gg CTT Tgg TTT Tgg AgC ggg TT	51	430	CA598941F CA598941R	CAT CAC CAA ggA ggA CA AAA gAA Cgg gAA gAg CC	48	405
CA597760F CA597760R	gTg CTg gCg ATg gTg CTC gCC gTT Cgg ggT TgT TgT	52	190	CA598151F CA598151R	gCg AgC CCT CCA CCA CAA Cgg CAA AgT AAT CAA TCA	42	402
CA598557F CA598557R	ATg ggg AAg AAg CAg gTg g TTg gTT TgA ACA Agg AAg A	43	441	CA598672F CA598672R	CAg Tgg gTg TCA ggA gTC T TgT gTT gTg TTg TgT TgT T	43	375
CA598691F CA598691R	AAg CCg AAg CAC TAg ATC C ACA TTC CAg AAA AAC ACg A	43	475	CA598755F CA598755R	AgC AAg CAA gCC gAA gCA CT Cgg gAA Agg AAA ACg gAg gA	51	358
CA599273F CA599273R	gCA gCT CCA gCg gCg CAg gC gCg gTg TAg gTg gTA Agg gT	54	146	CA599285F CA599285R	gCT CAC CAC CAC TAC TA ggA TgC CCg Cgg CCT TC	46	319
CA599115F CA599115R	CgT gCg ggC Agg Tgg ACT TgA CAT gCT gAT ggg gAA	52	252	CA599235F CA599235R	gAT ggC Tgg gCT ACT CTC T TTT ggA CCC CCg AAT TTT g	47	461
CA599277F CA599277R	gCT TTT TTC CCC TTC CTC Cg gCC CCT TTg AAT CAA TgT CC	50	552	CA598980F CA598980R	ATg AAC TgC TTC TgC TCC T TAg ATT TCg TAC TCT Tgg g	47	255
CA599015F CA599015R	CCA TAT CCT CTC CCA AgC TCC CAC CCA TTC TCA AAC	49	344	CA599119F CA599119R	CTC CCC AAA gCC CTA ACC AgC CAg gAA ggC gAA gAA g	53	380

**Table 6:** Similarity index (Jaccard's coefficient) between *Triticum aestivum* cultivars.

Population ID	PI178383	Izgi01	Sönmez2001	Harmankaya99	ES14	Aytin98
PI178383	1.000					
Izgi01	0.680*	1.000				
Sönmez2001	0.656*	0.692*	1.000			
Harmankaya99	0.692*	0.680*	0.725*	1.000		
ES14	0.682*	0.655*	0.686*	0.712*	1.000	
Aytin98	0.655*	0.622*	0.628*	0.655*	0.703*	1.000

*Genetically similar.
